# Supplementary material for: Inverse Design of Ultrathin Metamaterial Absorber
Source: Nanomaterials (Basel). 2025 Jul 1;15(13):1024. doi: 10.3390/nano15131024 (PMC12250792; doi:10.3390/nano15131024)
Supplement: Supplementary file 1 [file nanomaterials-15-01024-s001.zip › nanomaterials-3677983-supplementary.pdf]

Supplementary File

Inverse design of ultrathin metamaterial absorber

Eunbi Jang[1,†], Junghee Cho[2,†], Chanik Kang[3,*] and Haejun Chung [1,2,3,*]

[1] Department of Artificial Intelligence Semiconductor Engineering, Hanyang University, Seoul, 04763, South Korea

[2] Department of Electronic Engineering, Hanyang University, Seoul, 04763, South Korea

[3] Department of Artificial Intelligence, Hanyang University, Seoul, 04763, South Korea

† These authors contributed equally.

* Corresponding Author:

E-mail: chanik@hanyang.ac.kr, haejun@hanyang.ac.kr

Electromagnetic simulations were conducted using the finite-difference time-domain (FDTD) method implemented via the open-source software package MEEP [54]. The simulation domain was discretized with a uniform grid spacing of 125 μm, selected to achieve a balance between computational efficiency and numerical accuracy.

For two-dimensional (2D) simulations, Bloch periodic boundary conditions were applied along the horizontal (x-axis) direction, ensuring infinite periodicity horizontally. Perfectly matched layers (PML) were applied at the vertical (z-axis) boundaries to minimize reflections and simulate open boundary conditions in the propagation direction. In the three-dimensional (3D) simulations, Bloch periodic boundary conditions were employed along both horizontal axes (x- and y-axes), thereby emulating infinite periodicity in the transverse plane. Similarly, PML boundary conditions were applied along the propagation axis (z-axis) to effectively absorb outgoing waves and reduce reflections at simulation boundaries.

To ensure broadband performance, optimization was carried out over 50 uniformly spaced frequency points covering a range of 5–10 GHz, corresponding to free-space wavelengths from 60 mm to 30 mm. The optimized devices were verified to exhibit smooth spectral responses across the entire frequency band.

The investigated structure comprised an ultrathin conductive layer of Indium Tin Oxide (ITO), originally 25 nm in thickness. To maintain computational feasibility, this layer was modeled using a scaled effective thickness of 125 μm. This scaling approach preserved the relative optical

responses by proportionally adjusting permittivity and conductivity values [55]. The accuracy of this approximation was validated through comparisons with Transfer Matrix Method (TMM) simulations, showing numerical discrepancies of less than 0.23%. All simulations were performed at a target frequency of 7.5 GHz.

The complex permittivity values utilized for silicon (Si) and silicon dioxide (SiO_2) in the optimized design region were set as $\varepsilon_r = 11.9 + 0.0006i$ and $\varepsilon_r = 3.91 + 0.0015i$, respectively. These values were obtained by fitting experimentally measured dielectric properties over the operating frequency range using CST Studio Suite [56,57].

The initial design field was uniformly initialized at a value of 0.5, representing an intermediate (non-binary) material distribution. The degree of binarization was monitored during each iteration to ensure the convergence of the design toward physically realizable, binary structures.

Optimization was conducted using the Method of Moving Asymptotes (MMA), a gradient-based optimization algorithm based on the conservative convex separable approximation framework [58]. The MMA optimization was implemented using the open-source library NLopt [59], with convergence criteria defined by a gradient norm threshold below 10^{-3}. The optimization procedure involved multiple stages employing a continuation strategy that systematically increased the projection sharpness parameter (β) from an initial value of 4 to approximately 230 over nine stages, applying a multiplicative factor of 1.5. At each stage, the MMA algorithm was run for a maximum of 12 iterations.

All simulations and optimizations were performed on high-performance workstations equipped with AMD Ryzen Threadripper PRO 5995WX and 7995WX CPUs and 256 GB RAM. Parallel processing techniques were leveraged to enhance computational efficiency.
